# Acceptability of the Distress Thermometer and Problem List to community-based telephone cancer helpline operators, and to cancer patients and carers

**DOI:** 10.1186/1471-2407-11-46

**Published:** 2011-01-31

**Authors:** Karen L Hughes, Hilary Sargeant, Anna L Hawkes

**Affiliations:** 1 School of Nursing and Midwifery, University of Queensland, Brisbane, Australia; 2 Cancer Counselling Service, Cancer Council Queensland, Brisbane, Australia; 3 Viertel Centre for Research in Cancer Control, Cancer Council Queensland, Brisbane, Australia; 4 School of Public Health, Queensland University of Technology, Brisbane, Australia

## Abstract

**Background:**

Cancer can be a distressing experience for cancer patients and carers, impacting on psychological, social, physical and spiritual functioning. However, health professionals often fail to detect distress in their patients due to time constraints and a lack of experience. Also, with the focus on the patient, carer needs are often overlooked. This study investigated the acceptability of brief distress screening with the Distress Thermometer (DT) and Problem List (PL) to operators of a community-based telephone helpline, as well as to cancer patients and carers calling the service.

**Methods:**

Operators (n = 18) monitored usage of the DT and PL with callers (cancer patients/carers, >18 years, and English-speaking) from September-December 2006 (n = 666). The DT is a single item, 11-point scale to rate level of distress. The associated PL identifies the cause of distress.

**Results:**

The DT and PL were used on 90% of eligible callers, most providing valid responses. Benefits included having an objective, structured and consistent means for distress screening and triage to supportive care services. Reported challenges included apparent inappropriateness of the tools due to the nature of the call or level of caller distress, the DT numeric scale, and the level of operator training.

**Conclusions:**

We observed positive outcomes to using the DT and PL, although operators reported some challenges. Overcoming these challenges may improve distress screening particularly by less experienced clinicians, and further development of the PL items and DT scale may assist with administration. The DT and PL allow clinicians to direct/prioritise interventions or referrals, although ongoing training and support is critical in distress screening.

## Background

Cancer can be a distressing experience for cancer patients and carers, impacting on psychological, social, physical and spiritual functioning [1-9]. Between 10-50% of patients and carers suffer from ongoing, clinically significant psychological morbidity [6,8,10-12]. Health professionals often fail to detect distress in their patients [12] due to time constraints, and a lack of confidence in assessing distress and using psychometric instruments [13]. Also, with the focus on the patient, carer needs are often overlooked and only 50% of those with serious psychological distress will seek support [3,6].

In an effort to address barriers to distress screening, the National Comprehensive Cancer Network (NCCN) recommends the distress thermometer (DT), a single-item self-report measure of distress [14]. The DT is reportedly a brief, non-invasive, valid and acceptable alternative to longer psychometric instruments [15]. The Problem List (PL) can be used in addition to the DT to identify possible contributing factors, summarised into five categories: practical, family, emotional, spiritual, and physical. Although the DT and PL has been well-validated in numerous settings with various cancer groups [15-23] very little has been written on the acceptability of the measure.

The limited discussion indicates that in general, the DT is accepted and perceived as helpful and easy to complete by health professionals and cancer patients [17,24]. Three studies have indicated that use of the DT and PL promoted communication between the patient and health care team [17,24,25]. It has been suggested that the DT and PL give patients "permission" to open up on a wider range of issues [17,24]. Patients in one study noted that it was the first time they were asked about a number of issues [24]. Gessler et al. (2008) surveyed oncology and palliative care outpatients and found that 95% of respondents did not find completing the instrument upsetting in any way and 86% did not consider any changes were required to its current form [17]. Fulcher et al. (2007) reported that overall, patients were receptive to completing the tool with satisfaction ratings ("clinic's sensitivity to your needs") increasing from 88.1% to 92.6% following its introduction [24]. Further, health professionals indicated that the introduction of screening helped direct or prioritise interventions and referrals [24,25] and did not substantially burden the clinics or referral agencies [24]. Other attributes related to its acceptability include its speed, ease of administration, and its transparency [17,24].

However, some concerns with the DT and PL have also been identified. First, there is a concern that the options provided in the PL may not encompass all the critical issues experienced by cancer patients. Jacobsen et al. (2005) stated that the current format "does not provide an opportunity for patients to identify other potential sources of distress, such as unmet needs" [19]. Also, oncology and palliative outpatients have reported concern that the 0-10 scale is too simplistic and does not "give enough insight into issues" [17]. Some of these patients also suggested that the DT checklist was not comprehensive and required more items and symptom categories. Graves et al. (2007) tested a revised version of the PL indicating that information concerns and cognitive problems should also be assessed. Other suggested items included 'adjusting to my illness', and 'isolation/feeling alone' [26].

Difficulty in understanding the concept of distress has also been identified as a concern [17,24]. In one study a participant commented "what is the difference between distress and stress? I am stressed at work but not distressed" [17]. In a pilot study looking at introducing the DT and PL into standard practice at a radiation oncology clinic, nursing staff requested that a definition of distress be included with the questionnaire [24]. Finally both patients and health professionals have expressed discomfort addressing the emotive content of the PL [17,24]. A patient in one study stated that they felt uncomfortable acknowledging negative emotions [17]. Failure of nurses to utilise the DT has been linked to discomfort in discussing sexual and emotional issues with patients [24].

Cancer Council Queensland, a not-for-profit non-government organisation, recently utilised the DT and PL over a four month period to screen cancer patients and carers calling their telephone cancer helpline service operated by health professionals (nurses and allied health professionals). The current study investigates the acceptability of the tool to helpline operators and callers. Specifically, we report on how often the DT is utilised, the reported reasons for operators not utilising the DT, reasons for callers not responding to the DT, and reported benefits and challenges in utilising the tool from the operator's perspective. A content analysis of "other" problems - that is, problems that operators could not categorise into existing PL categories - is also provided.

## Methods

### Participants and Data Collection

#### Operators

All operators (n = 18) were asked to monitor their use of the DT and PL with eligible callers from September-December 2006. A convenience sample of seven operators was asked to respond to a brief, self-administered questionnaire: open-ended questions addressed benefits and challenges of utilising the DT and PL, administration over the telephone, usefulness in triaging, and frequency of use.

#### Callers

Over the four month study period (September-December 2006), 666 callers were recruited. Eligibility criteria included: diagnosed cancer patients or carer/support person, over 18 years of age, and English-speaking. Carer/support persons included immediate family members, relatives, or friends that were involved in the care of a diagnosed cancer patient (hereafter referred to as 'carers'). Operators were asked to record their reasons for not implementing the DT and PL, and any provided reasons why callers could not respond to the tool.

### Measurement

The DT is a single item, 11-point scale (0-10 with increasing distress) in a thermometer format used to rate level of distress. The associated PL asks respondents to respond to 34 items, although this study asked respondents to respond only to the specified categories (practical, family, emotional, spiritual/religious, and physical problems) rather than the individual items within each category. For instance "Have practical problems such as housing, finances, work, transport, or child care been a cause of your distress in the past week including today?" The DT and PL were modified for use over the telephone.

A recent meta-analysis reported the DT demonstrated 77.1% sensitivity and 66.1% specificity to detect cancer-related distress, and 80.9% sensitivity and 60.2% specificity to detect depression [20]. The screening performance of the DT is comparable to more rigorous and comprehensive criterion measures, including the Hospital Anxiety and Depression Scale (HADS), the Brief Symptom Inventory, the General Health Questionnaire-12, the Patient Health Questionnaire 9-item Depression Module; the Center for Epidemiological Studies-Depression Scale, and the European Organization for Research and Treatment of Cancer Quality of Life Questionnaire [15-19,21-23]. A cut-off score of four on the DT yields optimal sensitivity and specificity in comparison with "caseness," as established by the HADS [19,21,27]. This cut-off also identifies patients reporting high levels of physical, emotional, practical, and family problems [23,28-30]. The DT has been used to screen for distress across a range of cancer diagnoses including prostate cancer [31], newly diagnosed breast cancer [18,32], brain cancer [30]; mixed cancer diagnoses [19,33,34]; and for patients undergoing bone marrow transplant [23,35].

### Data Analysis

Descriptive analyses included frequencies, medians and interquartile ranges (IQR) to describe the callers (gender, carer versus patient status), call variables (operator, length of call) and DT utilisation and response. The impact of caller gender, patient versus carer status, length of call and operator on DT implementation and caller response was investigated using chi-square analyses and Mann-Whitney U-tests. Quantitative data were analysed using the 'Statistical Package for Social Sciences' (SPSS) 15.0 for Windows. Content analyses were conducted to identify common themes in the helpline operator open-ended or qualitative data.

### Ethical Approval

The study was approved by Griffith University Human Research Ethics Committee.

## Results

### Callers

Callers were a mixed-cancer group (n = 666) including cancer patients (n = 375) and carers (n = 291). The most commonly reported cancer types were breast (35%) and prostate (20%) cancer. The majority of callers were: cancer patients (56%) and women (78%). The median distress ratings were 5.0 (IQR = 4.0) and 6.0 (IQR = 3.0) for patients and carers, respectively (N.B., 16% of data was missing for this outcome). PL categories reported by callers are outlined in Table 1.

**Table 1:** Problem List (PL) categories reported by helpline callers (patients and carers)

	Patients n (%)	Carers n (%)
**PL Categories**		
Practical	89 (23.8)	63 (21.7)
Family	48 (12.8)	50 (17.2)
Emotional	170 (45.5)	159 (54.8)
Spiritual	5 (1.3)	4 (1.4)
Treatment	134 (35.8)	61 (21.0)
Symptoms	42 (11.2)	36 (12.4)
Other	91 (24.3)	62 (21.4)

### Use of the DT and PL

#### Predictors of use of the DT and PL

The DT and PL were utilised with 598 (90%) callers. Use of the DT by operators was not associated with patient versus carer status or gender of the caller (p ≥ 0.11), but was related to the length of the call (z = -8.41, p < 0.001). The median length of calls where the DT was asked (23.0 minutes, IQR = 20) was greater than when the DT was not asked (10.0 minutes, IQR = 10). There was a significant association between using the DT and operator (χ^2 ^= 50.4, df = 11, p < 0.001). Of the 12 operators taking at least 10 calls during the course of the study, three operators always utilised the DT, six operators used the DT over 90% of the time, and three operators reported using the tool 74-77% of the time.

##### Barriers to use of the DT and PL

Operators were questioned about reasons for not using the DT and PL and five broad themes emerged: (i) time or context restrictions (n_patients _= 10/35, 29%; n_carers _= 8/33, 24%); (ii) the caller's "reason" for calling was considered incongruous with distress screening (n_p _= 14, 40%; n_c _= 18, 54%); (iii) the caller was unreceptive or too ill to participate (n_p _= 6, 17%; n_c _= 2, 6%); (iv) communication difficulties (n_p _= 3, 8%; n_c _= 1, 3%); and (v) operator forgetting (n_p _= 2, 6%; n_c _= 4, 12%).

Time or context restrictions related to: caller being in a hurry (n_p _= 0; n_c _= 1); calling from work (n_p _= 2; n_c _= 2); needing to attend to something else (eg baby crying, husband waking, visitor arriving) (n_p _= 1; n_c _= 4); late for an appointment (n_p _= 1; n_c _= 0); or needing to end call for unspecified reasons (n_p _= 6; n_c _= 1). The callers' purpose or reason for the call was also considered before presenting the DT and PL. The following caller reasons were stated by operators to be inappropriate for DT administration: ringing for loaned medical equipment to be collected following the death of a patient (n_p _= 0; n_c _= 7); queries about the financial assistance program (n_p _= 2; n_c _= 1); queries about the wig service (n_p _= 4; n_c _= 0); ringing for other information (eg telephone contact number, publication) (n_p _= 3; n_c _= 6); call in relation to other bookings or appointments (eg book transport, change appointment with counselling service) (n_p _= 2; n_c _= 2); cancer free for several years and ringing about a new prosthesis or other information (n_p _= 3; n_c _= 0); spouse is palliative or recently deceased (n_p _= 0; n_c _= 2); and call was not cancer-related (n_p _= 2; n_c _= 0). Callers who were unresponsive or uncommunicative were also not asked the DT or PL in some instances (n_p _= 0; n_c _= 2). One caller (patient) expressed a desire not to answer a 'survey'. Also, patients who were confused, or too ill (eg feeling unwell from chemotherapy, short of breath with lung cancer) were also not asked to complete the DT and PL (n_p _= 5; n_c _= 0). Finally, operators reported not using the DT and PL if callers had limited English or difficulty hearing (n_p _= 3; n_c _= 1).

##### Caller response to use of the DT and PL

Of the 598 persons asked the DT, only 39 (6%) callers did not respond. Non-response to the DT was not related to cancer status or gender (p ≥ 0.28), or length of telephone call (p = 0.62). Of these 39 callers, 17 (43%) stated they didn't know, couldn't quantify ('couldn't put a number on it'), or described their distress ('says they are doing OK', 'the family say I'm very strong') (n_p _= 10; n_c _= 7). Seven callers (18%) became distressed and could not answer the question (eg, 'very tearful and upset') (n_p _= 6; n_c _= 1). Other reasons related to not being distressed or not feeling it was relevant to their needs (eg 'wanted to get off phone, called about a sex after treatment booklet', 'not distressed about son's cancer diagnosis, was 12 years ago') (n_p _= 1; n_c _= 1). One carer stated they had many concerns and were unable to rate, and one patient replied that other health issues were much worse. The final reasons given for callers not responding to the DT and PL included: illness (ie, 'very short of breath', n_p _= 1), leading the conversation in a different direction (ie, 'just started talking about something else', n_c _= 1), stating they were a 'spiritual healer' (n_p _= 1), and not understanding the question as English was not their first language (n_c _= 1).

#### Investigation of PL Categories

One-quarter (n = 153, 25%) of participants responses did not fit into the PL categories, that is they were categorised as 'other'. The most common problems identified in the 'other' option related to decision-making support or request for further information (n_p _= 28; n_c _= 10). Decision support was most often related to treatment. There were indications of adjustment to illness issues: 'reaction to diagnosis'; and 'coming to terms with diagnosis'. These numbers are likely an under-estimate as operator notes were not always clear or detailed. For instance a note indicating 'diagnosis and treatment' or 'prostate-specific antigen is climbing' may relate to a caller requiring additional information or decision support, but it may also relate to adjustment or emotional issues. Issues relating to grief and bereavement were also identified, particularly by the carers: 'end of life issues'; 'dealing with thought of husband dying'. Other types of loss were also mentioned: loss of independence (n_p _= 1; n_c _= 0) and loss of control (e.g., 'feels disempowered') (n_p _= 3; n_c _= 2). Loneliness or concern of being on their own was also mentioned by four cancer patients.

Problems related to different cancer stages were difficult to classify in existing PL categories, such as diagnosis (n_p _= 8; n_c _= 8), treatment (n_p _= 23; n_c _= 3), treatment outcomes and tests (n_p _= 7; n_c _= 3), progression or recurrence of disease (n_p _= 1; n_c _= 2), survivorship (n_p _= 0; n_c _= 1), and palliative (n_p _= 0; n_c _= 1). Some of these cancer stages were linked to specific emotions e.g., 'fear of chemo'. In terms of practical problems, difficulties with health professionals or the healthcare system (n_p _= 2; n_c _= 3) and financial concerns (n_p _= 1; n_c _= 1) were raised. Problems with parents and grandchildren were described as additional family concerns.

#### Operator response to use of the DT and PL

Operators identified several benefits to utilising the DT and PL with callers (see Table 2). Objectivity, structure and consistency were identified by three of the seven operators. Six operators described the instrument as a "useful opportunity" to probe the caller concerning emotion and coping. Operators also said the DT and PL were useful tools for identifying distress that was not overtly discernible. Utilising the DT score as a baseline for change was also discussed as a benefit (e.g., monitor change within and between calls; referral to counselling services).

**Table 2:** Key themes and illustrations related to helpline operator-perceived benefits and challenges of utilising the Distress Thermometer (DT) and Problem List PL), and utility in triage

Key themes	Illustrations
**Benefits**	

Objectivity and structure	"a formal record of the person's distress and problems are noted which ensures objectivity and the ability to triage ... to appropriate level of support"

Invites caller to share emotions	"reminds Operators to touch base with callers in regards to their emotional state/coping, as well as any other reason they called"; "it provides an opportunity to introduce the query about how callers are copying emotionally by 'normalising' the question"; "demonstrate to the caller our holistic approach to their care and support - we care about their emotional needs at this time"

Identifying distress not readily observed	"the rating may indicate to Helpline operator that the event is actually more stressful than what the person is portraying verbally"

Establishes baseline	"baseline ... to monitor the callers distress throughout and at end of call"

**Challenges**	

Awkward to administer	"It is sometimes difficult to find an appropriate moment during a call to ask the questions using the actual words in the tool"; "the DT feels clunky to use and is difficult to insert into a conversation when you have established rapport"

Inappropriate with particular callers	"the most difficult thing was keeping a distressed caller focused on giving an actual number rather than 'telling their story'"; "callers who do not call for emotional reasons, for example if they call ... for practical reasons such as a transport inquiry'; "there are some callers who are being supported well by family and friends and make it very clear that their call today is purely practical; "find asking those who are newly grieving (loss of loved one) to rate their distress very difficult...sometimes feels like asking them to rate their love for that loved-one"

Difficulty with the DT and PL scales	"some callers just don't get the number idea - they use words: 'no its all going alright' or 'a little bit', 'not really'"; "the language is hard for many men to identify with; ie the word 'distress' is not something they can associate with"; "proved difficult in the length of it [the PL] and not all callers wanted to go through it"

**Utility in triage**	

Objectivity and clear framework	"it gave me a clear framework to pursue with the caller which helped them to rate their level of distress and them identify what areas were causing the most distress. This then allowed me to assess gaps in knowledge and support which provided a basis for appropriate referral to services, support or information sources".

Same conclusion without the instrument	"in the main, I feel I probably made similar referrals prior to using the distress thermometer"

Clinical skills more valuable than DT	"you will have just explored how they are feeling, established a rapport, responded to their needs but are still required to ask this question...as an Operator I am not reliant on an instrument as blunt as this to determine how a person is going emotionally and what is the appropriate level of care for that person"

Not appropriate ALL the time	"it's not the this tool is inappropriate ALL of the time; in fact, there are times when it is an extremely valuable option to use with callers...what is inappropriate, given the range of calls operators take is to say that it MUST be used all the time"

Challenges with utilising the DT related to the perceived 'awkwardness' of administering the tool. One operator suggested that this concern reduced with experience and adequate training: "until the operator finds their own style of delivery and grows in ease through experience". Operators utilising the DT less often reported finding it difficult to use with very distressed or bereaved callers. They also found it more difficult to use with those calling with practical issues (e.g., transport inquiry), particularly if callers reported a strong support network..

Some operators mentioned concerns with administering a numeric scale over the telephone, although three operators stated they didn't have any, or had few, difficulties. General difficulties with a numeric scale and frustration with the length of the PL were stated. Certain groups were identified by operators as displaying particular difficulty: the elderly and callers from lower socio-economic backgrounds found it difficult to put "a value on their distress". Two operators said they had to "rephrase the scale/question" on occasion. One operator commented that men, in particular, had difficultly relating to the term "distress".

##### Caller triage using the DT and PL

When asked about its utility in triage, four operators said they found the DT and PL useful in making an appropriate decision concerning referral. Three operators mentioned the benefits of objectivity. Some operators stated that they would have formed the same conclusions without the instrument, with counselling skills identified as more valuable than the DT. It was also suggested that the DT was appropriate only on some occasions. Overall, the majority of operators (n = 5) 'mostly' used the instrument.

## Discussion

The DT and PL were used to screen for distress on the majority of eligible patients and carers calling the Cancer Council Queensland Cancer Helpline, and most callers provided valid responses to the instruments. A range of benefits to using the instruments were observed including having an objective, structured and consistent means for distress screening and triage to supportive care services. Reported challenges in using the DT and PL by operators included perceived inappropriateness of the instruments due to the nature of the call or level of caller distress, and the level of operator training.

Operators used the DT and PL on approximately 90% of all calls during the study period, and they were equally administered to male and female cancer patients and carers alike. The DT and PL were more likely to be asked on longer telephone calls, which may relate to the additional time required to administer the DT and PL, or the reason for the call as the perceived distress level and receptiveness of the caller resulted in briefer calls.

All of the operators used the DT and PL the majority of the time, although some operators experienced challenges with using the tools. There was a significant association between telephone operator and DT implementation, suggesting that less experienced operators may experience increased levels of personal discomfort or difficulty in using the tool. Failure to utilise the DT and PL by health professionals has been linked to discomfort discussing emotional or sexual issues [24]. Some operators in the present study found it difficult to incorporate the tools into their conversation, whilst some were uncomfortable or felt inexperienced with the use of the tools. One operator stated: "I believe failure to ask is usually because of my own discomfort in asking that particular caller. My experience is rarely is a caller unhappy to be asked. The more you ask, the easier it gets, more natural it becomes and the more you and the caller benefit". Therefore, ongoing training and support for operators is critical in the implementation of distress screening instruments, and additional support may be required for operators who identify their own discomfort in dealing with difficult emotions.

Operators suggested that there were occasions when administering the DT and PL seemed inappropriate such as when calls were of a practical nature (eg queries about services or equipment collection), or when callers were overtly distressed or newly bereaved. However, screening all callers for distress allows operators to direct or prioritise interventions or referrals [24,25], determine the exact level of distress for those who appear highly distressed, and provides operators with a rationale for assessing suicidality. Importantly, distress screening also allows operators to meet the needs of callers who otherwise may not receive emotional support. Finally, previous research has shown that patients are more satisfied with information services when emotional issues are discussed [24].

Operators also questioned whether the DT and PL provided additional information during the course of the call. Some felt that they were able to gauge how well a caller was coping through their own clinical judgement. In some cases this may have been true, such as when callers were quite explicit in describing their current coping and support needs. However, research has shown that health professionals do not assess patient distress accurately when it is not explicit [17,24,25]. Use of a distress screening tool in clinical practice ensures that all eligible callers are assessed and triaged, even if they are not showing overt signs of distress. It also allows for a baseline measurement of coping, another advantage identified by operators.

Some operators suggested that use of the term "distress" and the numeric scale were were confusing for some callers. One noted that certain callers (the elderly and those or low socio-economic status) appeared to have difficulty with the numeric scale and they had to rephrase the scale. This is an obvious concern when using a standardised measure with validated cut-offs. Some callers did not respond to the DT because they chose to describe their distress instead eg "doing OK". One way to overcome this barrier would be to match the numbers with descriptors of that level of distress. One operator said men were confused with the term "distress". Confusion or concern about whether patients understand this term has been discussed in other studies [17,24]. One group suggested a definition of distress be included in the questionnaire [24]. Other studies considered using other terms such as "mood" [33]. However, the term "distress" was selected by Roth et al. (1998) because it was thought to be less stigmatising and more acceptable to people [31]. It may be useful for further research to investigate whether certain sub-groups or people do indeed have difficulty with the numeric scale or terms used in the DT.

Content analysis of responses that did not fit into the existing PL categories indicated that additional categories may be useful. Decision support and information concerns were raised by some callers. Information concerns have been suggested as an additional PL category in other research [26] but extending this category to include decision making or decision support may also be useful. "Adjusting to illness" and "feeling alone" also appear to be valid additions to the PL as recommended by Graves et al. (2007) [26]. Other items worth considering in the Emotional Problems category relate to loss of independence or control, grief and bereavement. Practical problems relating to finance and the health system or health professionals could also be added. However, some considerations need to be taken into account with any changes to the PL. Firstly; it could be argued that a separate "carer" PL be developed. For instance, the listed physical problems only relate to patients. Also, there are likely to be issues specifically related to carer burden and bereavement. Secondly, additions to the PL have to be considered in its purpose as a "brief" screening tool. Each item should need to justify its inclusion and more research is required to identify whether the items are the most common problems experienced and importantly whether they assist appropriate referral. The PL may benefit from focus group research with cancer patients and carers.

The current study had a number of limitations. The DT and PL are predominantly utilised as self-administered instruments, although in the current study they were used in a cancer helpline context by telephone. This may reduce their generalisability to other settings. The operators did not always provide enough detail in their data collection to accurately categorise the "other" problems identified during PL administration. Whilst caller's response to the DT and PL were recorded by operators, callers were not specifically requested to detail their perception of the tools. Finally, limited socio-demographic information was collected from helpline callers as the purpose of the telephone call was to provide assistance rather than focus on data collection for research purposes.

## Conclusion

The DT and PL were used on the vast majority of eligible callers. As with previous studies [24,25], we observed a range of significant benefits to using the DT and PL including having an objective, structured and consistent way to screen for emotional distress and triage to supportive care services. However, operators reported some challenges. Overcoming these barriers may assist in widening the use of distress screening tools particularly by less experienced health professionals, and verbal descriptions for the DT values and further development of the PL items may assist with administration. Further research is required to identify appropriate items for a carers' PL.

Distress screening is key to managing cancer-related distress and meeting the supportive care needs of patients and carers. The DT and PL allow clinicians to direct or prioritise interventions or referrals appropriately, however ongoing training and support is critical in the implementation of distress screening. Also, additional support may be indicated for clinicians who identify their own discomfort in dealing with difficult emotions.

## Abbreviations

DT: Distress Thermometer; PL: Problem List; HADS: Hospital Anxiety and Depression Scale; IQR: Inter-Quartile Range.

## Competing interests

The authors declare that they have no competing interests.

## Authors' contributions

ALH developed the study protocol and was responsible for the implementation of the study. KLH drafted the manuscript and ALH and HS contributed to the final version. All authors read and approved the final manuscript.

## Pre-publication history

The pre-publication history for this paper can be accessed here:

http://www.biomedcentral.com/1471-2407/11/46/prepub
